# Survival impact of pre-treatment neutrophils on oropharyngeal and laryngeal cancer patients undergoing definitive radiotherapy

**DOI:** 10.1186/s12967-017-1268-7

**Published:** 2017-08-02

**Authors:** Whitney A. Sumner, William A. Stokes, Ayman Oweida, Kiersten L. Berggren, Jessica D. McDermott, David Raben, Diana Abbott, Bernard Jones, Gregory Gan, Sana D. Karam

**Affiliations:** 1 Department of Radiation Oncology, University of Colorado School of Medicine, Aurora, CO USA; 2 Department of Internal Medicine, Section of Radiation Oncology, University of New Mexico School of Medicine, Albuquerque, NM USA; 3 Division of Medical Oncology, Department of Medicine, University of Colorado School of Medicine, Aurora, USA; 4 Department of Biostatistics, University of Colorado School of Medicine, Aurora, CO USA

**Keywords:** Neutrophil, Chemoradiation, Larynx, Oropharynx, SCC

## Abstract

**Background:**

Squamous cell carcinoma of the head and neck (HNSCC) represents an array of disease processes with a generally unfavorable prognosis. Inflammation plays an important role in tumor development and response to therapy. We performed a retrospective analysis of HNSCC patients to explore the relationship of the lymphocyte and neutrophil counts, the neutrophil-to-lymphocyte ratio (NLR) overall survival (OS), cancer-specific survival (CSS), local control (LC) and distant control (DC).

**Materials/methods:**

All patients received definitive treatment for cancers of the oropharynx or larynx between 2006–2015. Neutrophil and lymphocyte counts were collected pre-, during-, and post-treatment. The correlations of patient, tumor, and biological factors to OS, CSS, LC and DC were assessed.

**Results:**

196 patients met our inclusion criteria; 171 patients were Stage III or IV. Median follow-up was 2.7 years. A higher neutrophil count at all treatment time points was predictive of poor OS with the pre-treatment neutrophil count and overall neutrophil nadir additionally predictive of DC. Higher pre-treatment and overall NLR correlated to worse OS and DC, respectively.

**Conclusion:**

A higher pre-treatment neutrophil count correlates to poor OS, CSS and DC. Lymphocyte counts were not found to impact survival or tumor control. Higher pre-treatment NLR is prognostic of poor OS.

**Electronic supplementary material:**

The online version of this article (doi:10.1186/s12967-017-1268-7) contains supplementary material, which is available to authorized users.

## Background

Despite advances in treatment, the prognosis of head and neck squamous cell carcinoma (HNSCC) patients remains poor. In the setting of definitive treatment, adding chemotherapy concurrently to radiotherapy improves progression-free survival (PFS), overall survival (OS), and organ preservation [1]. Despite advances, 5-year survival in HPV-negative and HPV-positive patients is 45 and 75%, respectively [1, 2]. For patients with locally advanced disease and heavy smoking history, the outcomes are far worse, approximating a survival rate of 25% at 5 years [3]. The presence of metastatic disease in HNSCC invariably portends a poorer prognosis, with treatment often limited to palliative regimens and an average survival of only 4–7 months [4].

Given the critical need to better understand these aggressive oncologic processes, there has been a concerted effort to identify features that impede, augment, or otherwise influence disease endpoints. Inflammation plays an important role in tumor development, progression, response to therapy and metastasis [5, 6]. Clinically, increasing evidence suggests that neutrophils, both those circulating in blood and those infiltrating tumors, may drive tumor progression and metastasis [7–10]. A number of studies have linked increased levels of circulating neutrophils with significantly worsened outcomes including OS in non-small cell lung cancer [11], cervical cancer [12], prostate cancer [13, 14], gastric cancers [15–17], bladder cancer [18] and colorectal cancer [19]. Several reports have recently detailed the role of both circulating [20, 21] and tumor infiltrating neutrophils [22] as predictors of survival in HNSCC as well and their role in promoting of distant metastasis [23].

Additional prognostic value has been shown with the neutrophil-to-lymphocyte ratio (NLR) with higher values portending poor survival [24–26]. Circulating lymphocyte counts have been independently shown to improve OS in malignancy, with Rachidi et al. specifically highlighting their prognostic value in HNSCC [24, 28].

Here, we investigate the prognostic value of circulating neutrophil and lymphocyte counts as well as NLR at multiple time points on OS, cancer-specific survival (CSS), local control (LC) and distant control (DC) in patients diagnosed with oropharyngeal and laryngeal cancer.

## Methods

### Data source and patient selection

We identified 356 patients with squamous cell carcinoma of the oropharynx or larynx diagnosed between 2006 and 2015 with available follow up and treatment outcomes who were treated with curative-intent radiation therapy (RT) within the University of Colorado Hospital system (n = 336) and the University of New Mexico Hospital (n = 20) systems. In order to ensure adequate assessment of response to treatment without potential confounding of post-treatment changes on imaging scans, patients were excluded if they had fewer than 6 months of follow up from completion of treatment [29]. Patients must have received minimum of two blood draws for complete blood count with differential during the period stretching from 1 month prior to initiation of treatment, including chemotherapy and radiation, through 1 month post-treatment. Additionally, patients were excluded if the radiation treatment course was prolonged by greater than 1 week past the planned completion date. A total of 196 patients met these criteria.

### Patient demographics and treatment variables

All patients received external beam radiation to a minimum dose of 54 Gray (Gy) (range 54–70 Gy, median 69.3 Gy, standard deviation (SD) 3.98 Gy) using 1.8–2.25 Gy/fraction. A total of 5 patients received less than 60 Gy. The median number of radiation treatments was 33 (range 27–35) over an average of 48 days. Potentially relevant patient and treatment characteristics were included. Age at diagnosis was analyzed categorically based on the approximate median age of 60 years. Race was categorized as white or other. Performance status was categorized according to the Karnofsky Performance Score (KPS) as ≤80 or >80 [30]. T-classification and N-classification were recorded according to the American Joint Committee on Cancer (AJCC) classification 2002 and 2010 editions based on date of diagnosis. Tobacco use was divided into <10 or ≥10 pack years [31]. Patients were considered post-operative if they had received any of the following: subtotal resection, gross total resection, or neck dissection (ipsilateral or bilateral). Chemotherapy was analyzed as a binary variable (yes/no). Additional analyses included steroid use as a binary variable with >100 mg oral equivalents of dexamethasone versus <100 mg and chemotherapy regimens stratified into none, cisplatin weekly, cisplatin every 3 weeks (q3), carboplatin plus paclitaxel and cisplatin plus cetuximab.

The cohort was divided into tertiles by neutrophil count (tertile 1: ≤3.2 × 1000 mm^3^ (n = 65), tertile 2: 3.2–4.4 × 1000 mm^3^ (n = 66) and tertile 3: >4.4 × 1000 mm^3^ (n = 65), and Kaplan–Meier analysis was performed. For multivariate analysis (MVA), the neutrophil and lymphocyte counts were assessed as continuous variables. The primary endpoints were OS, CSS, LC and DC. Survival was determined from the date of diagnosis to the date of death or most recent follow-up. Cancer-specific survival was defined as cancer survival unrelated to other causes of death. Local failure included recurrence or progression in the primary tumor bed and surrounding tissues or regional lymph nodes. Distant failure was defined as a site of disease outside of the tumor bed and regional lymph nodes that was not present during initial therapy.

### Definition of white blood cell counts

Three time-periods were defined during the treatment course for collection of absolute neutrophil count (ANC) and absolute lymphocyte count (ALC): (1) 1 month prior to initiation of any treatment (pre-treatment), (2) 1 week to 1 month post-treatment (post-treatment) and (3) containing all time periods from pre-treatment to post-treatment (overall). For the collection of pre-treatment counts, values were taken from the day of treatment initiation whenever possible (190 of 196 patients). For the remaining time points, the lowest value, or nadir, of the ANC and ALC during each respective time period were collected. Analyses were performed at each time period for the ANC, ALC and NLR. The data was then combined over all time-periods and the lowest ANC and ALC observed were selected to represent the overall nadir for analysis. Additionally, the absolute difference in neutrophil count from pre-treatment to post-treatment was collected for analysis.

### Statistical analysis

All statistical analyses were performed using SPSS V23.0 (SPSS Inc., Chicago, IL). Pearson Chi square tests were used to assess associations between categorical variables and blood counts. Median follow up was calculated using the reverse KM method [31]. OS, CSS, LC and DC were first examined using the KM method. Univariate survival analysis (UVA) was performed with the log-rank test and unadjusted Cox proportional hazards models to estimate hazard ratios (HR), with HR >1 corresponding to worse OS, CSS, LC and DC. Patient and clinical variables were selected a priori. Multivariate Cox regression analysis was performed on all blood counts found to be significant (*p* < 0.05) on UVA using OS, CSS, LC and DC as outcomes with a significance level of *p* < 0.05.

## Results

Among our 196 patients, median follow up was 2.7 years (range 0.5–10.8). Median population age was 58 years (range 27–81 years). 171 patients were Stage III or IV. For patients who received chemotherapy (n = 182), regimens included cisplatin (weekly or q3 weeks), carboplatin plus paclitaxel, cetuximab or cisplatin plus cetuximab. In total, 30 patients (15.3%) experienced local failure and 21 patients (10.7%) experienced distant metastasis. Of local failures, 5 patients experienced disease progression in the local tumor bed, while 25 patients experienced a complete response to therapy followed by local recurrence. Of the 21 metastases, 16 were found in the lungs, 4 were found in bone and 1 patient experienced failure in both the lung and bone. The average times to progression, local recurrence, and distant failure were 0.60 years, 1.44 years and 1.53, respectively. There were 7 HPV positive patients with laryngeal primaries. The remaining 92 patients were oropharyngeal primaries. Of the oropharyngeal patients, 28/145 had unknown HPV status. Patient and treatment characteristics are presented in Table 1.

**Table 1:** Patient characteristics

	All patients (n = 196)	
	#	%
Age at diagnosis, years		
<60	120	61.2
>60	76	38.8
Primary site		
Oropharynx	145	74.0
Larynx	51	26.0
Gender		
Male	171	87.2
Female	25	12.8
Race		
White	171	25
Other	87.2	12.8
Tobacco use (pack years)		
<10	106	54.1
≥10	90	45.9
Karnofsky performance score		
>80	140	71.4
≤80	56	28.6
T-stage		
T1	102	52
T2	56	28.6
T3	30	15.3
T4	8	4.1
N-stage		
N0–1	61	31.1
N2a–2b	91	46.4
N2c–3	44	22.4
Post-operative radiation		
Yes	48	24.5
No	148	75.5
HPV		
Negative	51	26
Positive	99	50.5
Unknown	46	23.5
Chemotherapy		
None	14	7.1
Cisplatin, weekly	38	19.4
Cisplatin, q3 weeks	39	19.9
Carboplatin + paclitaxel	38	19.4
Cetuximab	57	29.1
Cisplatin + cetuximab	10	5.1
Overall survival, years		
Median	2.7	
Mean	3.39	
95% CI	3.04–3.75	

Analysis of the absolute and relative difference between the pre-treatment and post-treatment neutrophil counts for each patient revealed no significant difference in any endpoints (Additional file 1: Table S1). The majority of patients (68%) experienced a decline in neutrophil count, though the average absolute change in neutrophil count from pre-treatment to post-treatment was <1 × 1000 mm^3^. Six percent of patients experienced no change in neutrophil count, while 26% of patients experienced an increase in neutrophil count from pre-treatment to post-treatment with an average gain of 1.5 × 1000 mm^3^.

### Analysis of patient characteristics

Univariate analysis *p* values of each patient characteristic for all endpoints are presented in Table 2. OS was significantly worse for patients with tobacco use ≥10 pack-years, T-3–4 and HPV-negative status. CSS was worse among those with KPS ≤80, and T-classification 3–4. A decrease in LC was seen in patients with T-classification 3–4 and HPV-negative status. There were no factors that independently correlated to decreased DC. Factors that did not influence any endpoints include age, primary site, gender, race, nodal status, post-operative status and receipt of chemotherapy. Pearson Chi square analysis of chemotherapy regimens with overall neutrophil nadir did not indicate a significant correlation (0.039, *p* = 0.60). Similarly, no correlation was observed between receipt of steroids and overall neutrophil nadir during the treatment period (*r* = 0.18, *p* = 0.14). Univariate analysis of RT dose as a continuous variable did not reveal a significant correlation with OS or CSS (HR, 1.00; 95% CI 1.00–1.01; p = 0.41 and HR, 1.00; 95% CI 0.99–1.00; p = 0.91, respectively).

**Table 2:** Univariate analysis of patient characteristics, p values

	Overall survival	Cancer-specific survival	Local control	Distant control
Age at diagnosis, years				
<60 vs. ≥60	HR, 1.72; 95% CI 0.92–3.20; *p* = 0.09	HR, 0.83; 95% CI 0.48–1.44; *p* = 0.51	HR, 1.34; 95% CI 0.64–2.79; *p* = 0.44	HR, 1.03; 95% CI 0.43–2.48; *p* = 0.95
Primary site				
Oropharynx vs larynx	HR, 1.56; 95% CI 0.81–2.99; *p* = 0.18	HR, 0.84; 95% CI 0.45–1.55; *p* = 0.57	HR, 1.82; 95% CI 0.86–3.85; *p* = 0.12	HR, 1.20; 95% CI 0.47–3.10; *p* = 0.71
Gender				
M vs F	HR, 0.68; 95% CI 0.24–1.91; *p* = 0.46	HR, 0.59; 95% CI 0.24–1.48; *p* = 0.26	HR, 0.69; 95% CI 0.21–2.29; *p* = 0.55	HR, 0.62; 95% CI 0.15–2.68; *p* = 0.53
Race				
White vs other	HR, 1.24; 95% CI 0.52–2.95; *p* = 0.63	HR, 0.98; 95% CI 0.45–2.18; *p* = 0.98	HR, 1.24; 95% CI 0.5202.95; *p* = 0.59	HR, 0.37; 95% CI 0.05–2.74; *p* = 0.33
Tobacco use (pack years)				
<10 vs. ≥10	*HR*, *2.97*; *95% CI 1.48*–*5.97*; *p* < *0.01*	HR, 1.31; 95% CI 0.78–2.21; *p* = 0.31	HR, 1.92; 95% CI 0.89–4.13; *p* = 0.10	HR, 1.41; 95% CI 0.59–3.34; *p* = 0.44
Karnofsky performance score				
>80 vs. ≤80	HR, 0.97; 95% CI 0.50–1.89; *p* = 0.94	*HR*, *0.48*; *95% CI 0.25*–*0.92*; *p* = *0.03*	HR, 1.23; 95% CI 0.58–2.63; *p* = 0.59	HR, 1.16; 95% CI 0.47–2.83; *p* = 0.75
T-stage				
T1/2 vs T3/4	*HR*, *2.83*; *95% CI 1.47*–*5.44*; *p* < *0.01*	*HR*, *6.47*; *95% CI 3.34*–*12.52*; *p* < *0.01*	*HR*, *2.21*; *95% CI 1.42*–*3.44*; *p* < *0.01*	HR, 1.53; 95% CI 0.65–3.64; *p* = 0.33
N-stage				
N0-1 vs N2/3	HR, 0.98; 95% CI 0.51–1.89; *p* = 0.96	HR, 1.65; 95% CI 0.90–3.02; *p* = 0.10	HR, 0.79; 95% CI 0.37–1.67; *p* = 0.53	HR, 1.26; 95% CI 0.49–3.26; *p* = 0.63
Post-operative radiation				
Yes vs No	HR, 0.70; 95% CI 0.32–1.52; *p* = 0.37	HR, 1.01; 95% CI 0.56–1.82; *p* = 0.97	HR, 0.47; 95% CI 0.16–1.36; *p* = 0.17	HR, 1.70; 95% CI 0.70–4.12; *p* = 0.24
HPV				
Positive vs negative	*HR*, *0.32*; *95% CI 0.15*–*0.67*; *p* < *0.01*	HR, 1.00; 95% CI 0.55–1.84; *p* = 0.98	*HR*, *0.25*; *95% CI 0.10*–*0.62*; *p* < *0.01*	HR, 0.49; 95% CI 0.19–1.27; *p* = 0.14
Chemotherapy (v none)				
Yes	HR, 1.43; 95% CI 0.34–5.94; *p* = 0.62	HR, 1.45; 95% CI 0.45	HR, 1.19; 95% CI 0.28–5.03; *p* = 0.81	HR, 1.90; 95% CI 0.25–14.19; *p* = 0.50
Cisplatin, weekly	HR, 1.14; 95% CI 0.23–5.64; *p* = 0.88	HR, 1.21; 95% CI 0.33	HR, 1.12; 95% CI 0.22–5.77; *p* = 0.90	HR, 3.57; 95% CI 0.44–29.23; *p* = 0.24
Cisplatin, bolus	HR, 1.25; 95% CI 0.25–6.23; *p* = 0.78	HR, 2.74; 95% CI 0.80	HR, 0.82; 95% CI 0.15–4.59; *p* = 0.82	HR, 2.20; 95% CI 0.24–19.85; *p* = 0.48
Carboplatin	HR, 1.41; 95% CI 0.30–6.59; *p* = 0.66	HR, 1.15; 95% CI 0.31	HR, 1.79; 95% CI 0.39–8.33; *p* = 0.46	HR, 0.82; 95% CI 0.07–9.11; *p* = 0.87
Cetuximab	HR, 1.91; 95% CI 0.44–8.31; *p* = 0.39	HR, 1.18; 95% CI 0.34	HR, 1.09; 95% CI 0.23–5.13; *p* = 0.91	HR, 1.70; 95% CI 0.20–14.11; *p* = 0.63
Cetuximab and cisplatin	HR, 0.51; 95% CI 0.05–5.71; *p* = 0.59	HR, 1.13; 95% CI 0.23	HR, 1.00; 95% CI 0.13–7.77; *p* = 1.00	HR, 1.36; 95% CI 0.09–21.88; *p* = 0.83

### Factors that influence overall survival

**Fig. 1:**
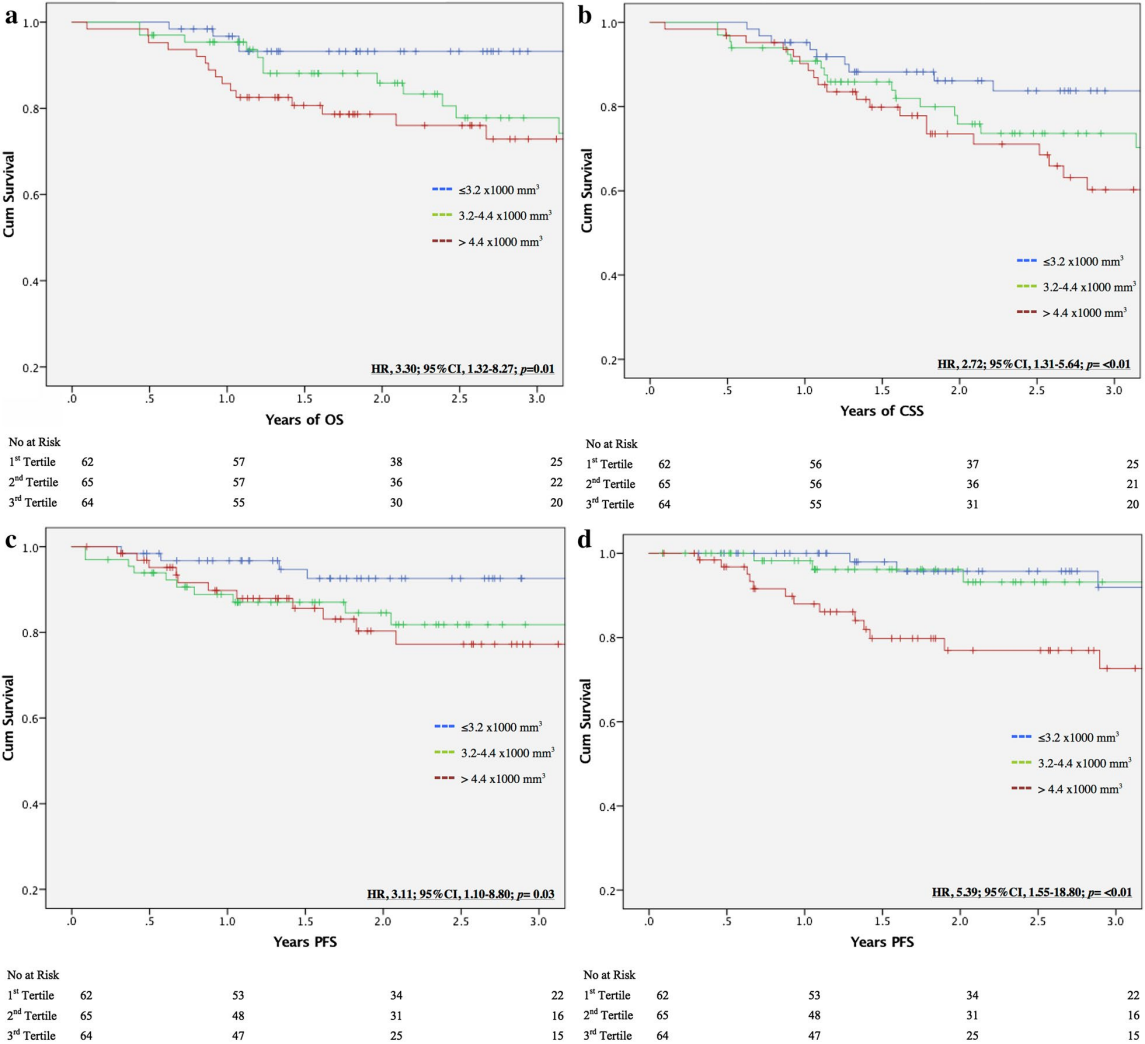
Unadjusted Kaplan–Meier curve demonstrating **a** overall survival, **b** cancer-specific survival, **c** local control and **d** distant control for pre-treatment neutrophil counts of ≤3.2 × 1000 mm^3^ (*blue*), 3.2–4.4 × 1000 mm^3^ (*green*) and >4.4 × 1000 mm^3^ (*red*). Cox-regression UVA reflects pre-treatment neutrophil count as a continuous variable

Two-year OS progressively diminished with higher pre-treatment neutrophil nadir with tertiles 1, 2 and 3 at 93.0, 86.0 and 78.5%, respectively (p = 0.03). These results are shown in Fig. 1a. On both UVA and MVA, all neutrophil time-points (pre-treatment, post-treatment and overall nadir) correlated a higher neutrophil count to worse OS (Table 3). The pre-treatment NLR was additionally predictive of OS on both UVA (HR, 1.07; 95% CI 1.01–1.14; *p* = 0.02) (Table 3) and MVA (HR, 1.09; 95% CI 1.01–1.17; *p* = 0.03) (Table 4). A summary of UVA for all outcome measures is shown in Table 5. The MVA for pre-treatment neutrophil count and OS including all patient characteristics is shown in Additional file 1: Table S2.

**Table 3:** Univariate analysis of neutrophil count, lymphocyte count and NLR at each time point

	Neutrophil count			Lymphocyte count			NLR		
	HR	95% CI	*p*	HR	95% CI	*p*	HR	95% CI	*p*
Overall nadir (n = 196)									
Overall survival	1.27	1.09–1.47	*<0.01*	1.60	0.93–2.76	0.09	1.01	0.98–1.05	0.51
Cancer-specific survival	1.14	0.99–1.31	0.07	0.86	0.45–1.62	0.63	1.01	0.98–1.04	0.50
Local control	1.23	1.04–1.46	*0.02*	1.53	0.83–2.83	0.18	1.00	0.95–1.05	0.92
Distant control	1.18	0.95–1.49	0.14	0.38	0.08–1.75	0.21	1.05	1.01–1.10	*0.01*
Pre treatment (n = 194)									
Overall survival	1.21	1.02–1.44	*0.03*	1.03	0.68–1.55	0.89	1.07	1.01–1.14	*0.02*
Cancer-specific survival	1.22	1.05–1.41	*<0.01*	1.18	0.85–1.63	0.33	1.04	0.98–1.11	0.17
Local control	1.16	0.95–1.42	0.15	1.41	0.97–2.05	0.07	1.05	0.96–1.14	0.31
Distant control	1.37	1.09–1.47	*<0.01*	1.45	0.95–2.21	0.09	1.00	0.89–1.13	1.00
Post treatment (n = 136)									
Overall survival	1.29	1.06–1.56	*0.01*	1.10	0.36–3.35	0.87	1.03	0.98–1.08	0.31
Cancer-specific survival	1.25	0.99–1.57	0.06	0.47	0.12–1.89	0.29	1.04	0.99–1.10	0.10
Local control	1.47	1.16–1.86	*<0.01*	1.42	0.39–5.12	0.60	1.04	0.99–1.09	0.17
Distant control	1.18	0.88–1.57	0.27	1.65	0.47–5.81	0.44	0.98	0.91–1.06	0.67

**Table 4:** Multivariate analysis of hematologic factors significant on UVA

	Multivariate Analysis		
	HR	95% CI	*p*
Overall neutrophil nadir (n = 196)			
Overall survival	1.36	1.12–1.66	<0.01
Distant control	1.32	1.01–1.73	0.05
Pre treatment neutrophil count (n = 194)			
Overall survival	1.28	1.02–1.61	0.03
Cancer-specific survival	1.32	1.10–1.59	<0.01
Distant control	1.48	1.13–1.95	<0.01
Post treatment neutrophil count (n = 136)			
Overall survival	1.35	1.04–1.74	0.02
Local control	1.58	1.21–2.07	<0.01
Overall NLR			
Distant control	1.06	1.01–1.11	0.02
Pre treatment NLR			
Overall survival	1.09	1.01–1.17	0.03

**Table 5:** Summary of prognostic factors on univariate analysis

	Overall survival	Cancer-specific survival	Local control	Distant control
Pre-treatment neutrophil count	•	•		•
Post-treatment neutrophil count	•		•	
Overall neutrophil nadir	•		•	
Pre-treatment lymphocyte count				
Post-treatment lymphocyte count				
Overall lymphocyte nadir				
Pre-treatment NLR	•			
Post-treatment NLR				
Overall NLR				•

• Denotes *p* ≤ 0.05 for the respective parameter on cox-regression

### Factors that influence cancer-specific and disease-free survival

Similar to the OS findings, 2-year CSS progressively diminished with higher pre-treatment neutrophil nadir with tertiles 1, 2 and 3 at 86.0, 76.0 and 73.5%, respectively (p = 0.009) Fig. 1b). This was consistent on MVA with pre-treatment neutrophil count portending poor CSS (HR, 1.32; 95% CI 1.10–1.59; *p* = <0.01) (Table 4).

### Factors that influence local control

Pre-treatment neutrophil count in tertiles 1 through 3 indicated that 2-year LC was 92.5, 84.5 and 82.5%, respectively, which was not statistically significant (Fig. 1c). On both UVA and MVA, a higher post-treatment neutrophil count correlated to worse LC (HR, 1.47; 95% CI 1.16–1.86; *p* = <0.01 (Table 3) and HR, 1.58; 95% CI 1.21–2.07; *p* = <0.01 (Table 4), respectively).

### Factors that influence distant control

Between tertiles 1 and 2, 2-year DC for pre-treatment neutrophil count was 96 and 93%, respectively, while tertile 3 was 79% (p = <0.01) (Fig. 1d). On MVA, a higher pre-treatment neutrophil count was also shown to predict worse DC (Table 4). On both UVA and MVA, a higher overall NLR correlated to worse DC (Tables 3, 4, respectively).

## Discussion

To date, the clinical significance of circulating blood counts in patients with HNSCC continues to be defined. Here we show that the circulating neutrophil count and NLR may each be independent prognostic indicators in HNSCC patients. Most impressive among these findings is that neutrophil counts were found to correlate with all outcome measures with a higher pre-treatment neutrophil count emerging as a particularly strong portent of worse OS, CSS and DC.

These findings add to the growing body of evidence that a higher pre-treatment neutrophil count portends adverse survival and treatment outcomes in cancer patients [13, 17–19] and align with those who have linked baseline neutrophil count and neutrophil nadir to OS in non-small cell lung cancer and ovarian cancer [32]. In addition, these findings now have a prospective correlate with the recent release of the LAP 07 trial demonstrating that neutrophilia portends poor OS in pancreatic carcinoma [33]. While prior studies have noted a correlation with OS and neutrophil count in HNSCC, we present the first to directly correlate the neutrophil count to CSS, LC and DC.

In conjunction with this intriguing neutrophil data, we also support prior evidence demonstrating poor survival in HNSCC patients with high pre-treatment NLR [24–27, 34–36]. While this confirmation is encouraging, we did not see a correlation between NLR and CSS or tumor control as was seen prominently with neutrophil counts.

In contrast with the current literature, we did not find the lymphocyte count to be a prognostic indicator for survival. While several studies have shown higher circulating lymphocyte counts to improve OS [24, 28], there are a number of reasons why these counts could be unreliable with the most critical question being whether or not all circulating lymphocytes are functional in the setting of malignancy. Lymphocyte exhaustion and dysfunction in malignancy have been well described in HNSCC [37, 38], possibly stemming from impairment of natural killer cell function and dysfunction in antigen presentation. This suggests that the circulating lymphocyte count may have variable significance and thus limit the prognostic value of the ALC.

Despite the understanding that systemic chemotherapy has the potential to decrease circulating WBC, this was not deemed a significant factor in our analysis. It is additionally necessary to state that our analysis does not serve to implicate the therapeutic regimens as the cause of leukocyte changes, but rather to form an initial understanding of the prognostic importance of the leukocyte count irrespective of the cause.

Developing a firmer grasp on prognostic factors and their reliability is particularly critical as approximately 60% of HNSCC patients present with locally advanced disease [39]. Despite recent improvements, the majority of patients with locally advanced disease go on to develop local and/or regional recurrences and approximately one in four develop distant metastases [40, 41]. According to our findings, neutrophil counts and neutrophil-associated inflammation represent promising avenues for exploration, particularly with accumulating, though still controversial, evidence suggesting that neutrophils have the capacity to facilitate tumor growth and metastasis. Specifically in HNSCC, Trellakis et al. showed that tumor infiltration with high numbers of polymorphonuclear lymphocytes (PMNs) in advanced HNSCC is a poor prognostic indicator for survival [10].

While originally assumed to be a harmless indicator of a failed immune response, neutrophils may in fact have a detrimental effect by supporting the development, growth, and progression of tumors [42, 43]. One hypothesis asserts that immune cells are recruited to potential metastatic sites, where they secrete factors that facilitate tumor cell survival and growth, creating a permissive growth environment for tumor cells [43]. Once at the pre-metastatic site, bone marrow-derived cells secrete factors that facilitate tumor cell survival and growth [34, 35]. Neutrophils in particular have been implicated in promoting tumor growth and angiogenesis via the secretion of pro-angiogenic factors including MMP9 and VEGF [36].

The connection of neutrophils with distant metastasis has additionally been attributed to tumor “piggybacking” on neutrophils to traverse the endothelium [44, 45]. Further, preferential blockade of neutrophil chemotactic factors including IL-8 has been shown to promote extracellular matrix (ECM) degradation, a critical step in allowing tumor cells to access the vasculature [44]. While the precise mechanism of neutrophil-assisted metastasis is still unclear, evidence suggests that the presence of cytokines including TGF-β and IFN-β may influence neutrophil signaling to serve a pro-metastatic function via immunosuppression [45]. This is supported by a study showing that neutrophils isolated from spleens of tumor-bearing mice inhibited the generation of effector CD8+ T-cells [46]. Finally, neutrophils in animal models have been shown during high-inflammatory states to produce extracellular DNA webs, known as neutrophil extracellular traps (NETs), that neutralize antigenic cells, while their presence in the setting of malignant cells predicts an increased risk of distant metastases [9].

## Conclusions

In summary, circulating blood counts represent easily measured, reproducible, and objective clinical marker of systemic inflammation in cancer patients. Our results demonstrate that the pre-treatment neutrophil count can serve as an independent prognostic indicator of survival and distant control in oropharyngeal and laryngeal cancer patients undergoing definitive treatment. Lymphocyte count is not an indicator of survival. The pre-treatment NLR, however, does correlate to OS. There are inherent limitations to our analysis including the retrospective approach of data collection, combination of two HNSCC sub-sites, heterogeneous response to therapy and the potential for unknown confounders impacting patient outcomes. While further studies are certainly needed to validate circulating and infiltrating markers for risk stratification and response to therapy in HNSCC, these findings augment our current understanding of the role of neutrophils and lymphocytes in tumor metastasis and survival.

## Additional file

**Additional file 1: Table S1.** Outcomes by raw difference in neutrophil count from pre-treatment to post-treatment (n = 196). **Table S2.** Multivariate predictors of overall survival.

## Abbreviations

OS: overall survival
CSS: cancer-specific survival
LC: local control
DC: distant control
DFS: disease-free survival
TCGA: the cancer genome atlas
TIN: tumor-infiltrating neutrophils
KM: Kaplan–Meier
UVA: univariate
MVA: multivariate
HNSCC: head and neck squamous cell carcinoma
KPS: Karnofsky performance status
ANC: absolute neutrophil count
ALC: absolute neutrophil count
NLR: neutrophil-to-lymphocyte ratio
HR: hazard ratio
AJCC: American Joint Committee on Cancer
ECM: extracellular matrix
