# Supplementary material for: Survival impact of pre-treatment neutrophils on oropharyngeal and laryngeal cancer patients undergoing definitive radiotherapy
Source: J Transl Med. 2017 Aug 2;15:168. doi: 10.1186/s12967-017-1268-7 (PMC5539641; doi:10.1186/s12967-017-1268-7)
Supplement: Supplementary file 1 — Additional file 1: Table S1. Outcomes by raw difference in neutrophil count from pre-treatment to post-treatment (n = 196). Table S2. Multivariate predictors of overall survival. [file 12967_2017_1268_MOESM1_ESM.pdf]

Supplementary 1. Outcomes by Raw Difference in Neutrophil Count from Pre-Treatment to Post-Treatment (n=196)

Outcome Measure	Univariate Analysis		
	HR	95%CI	*p*
Overall Survival			
Continuous	0.97	0.78-1.21	0.78
Cancer Specific Survival			
Continuous	1.05	0.82-1.35	0.68
Local Control			
Continuous	0.84	0.66-1.07	0.16
Distant Control			
Continuous	1.07	0.81-1.41	0.66

Supplementary Table 2. Multivariate Predictors of Overall Survival

	Multivariate		
**Variable**	**HR**	**95% CI**	
Age at Diagnosis			
≤ 60	1		
> 60	0.83	0.39-1.74	0.62
Primary Site			
Oropharynx	1		
Larynx	0.74	0.28-1.98	0.55
Gender			
Male	1		
Female	0.38	0.12-1.24	0.11
Race			
White	1		
Other	1.08	0.39-2.96	0.89
Tobacco Use (pack years)			
< 10	1		
≥ 10	2.4	1.08-5.34	0.03
Karnofsky Performance Score			
> 80	1		
≤ 80	1	0.46-2.19	1.00
T-stage			
T1-2	1		
T3	2.02	0.92-4.44	0.08
T4	3.9	1.60-9.48	<0.01
N-stage			
N0-1	1		
N2a-2b	1.14	0.47-2.73	0.78
N2c-3	0.23	0.06-0.86	0.03
Post-Operative Radiation			
Yes	1		
No	0.83	0.34-2.00	0.67
HPV			
Negative	1		
Positive	0.31	0.12-0.77	0.01
Unknown	0.34	0.12-0.93	0.04
Chemotherapy			
No	1		
Yes	4.93	0.96-25.45	0.06
Pre-Treatment Neutrophil Count			
Continuous	1.28	1.02-1.61	0.03

Abbreviations: HR, hazard ratio; CI, confidence interval
